# Radiation Induced Chromatin Conformation Changes Analysed by Fluorescent Localization Microscopy, Statistical Physics, and Graph Theory

**DOI:** 10.1371/journal.pone.0128555

**Published:** 2015-06-04

**Authors:** Yang Zhang, Gabriell Máté, Patrick Müller, Sabina Hillebrandt, Matthias Krufczik, Margund Bach, Rainer Kaufmann, Michael Hausmann, Dieter W. Heermann

**Affiliations:** 1 Institute for Theoretical Physics, Heidelberg University, Philosophenweg 19, 69120, Heidelberg, Germany; 2 Kirchhoff-Institute for Physics, Heidelberg University, Im Neuenheimer Feld 227, 69120, Heidelberg, Germany; Schulze Center for Novel Therapeutics, Mayo Clinic, UNITED STATES

## Abstract

It has been well established that the architecture of chromatin in cell nuclei is not random but functionally correlated. Chromatin damage caused by ionizing radiation raises complex repair machineries. This is accompanied by local chromatin rearrangements and structural changes which may for instance improve the accessibility of damaged sites for repair protein complexes. Using stably transfected HeLa cells expressing either green fluorescent protein (GFP) labelled histone H2B or yellow fluorescent protein (YFP) labelled histone H2A, we investigated the positioning of individual histone proteins in cell nuclei by means of high resolution localization microscopy (Spectral Position Determination Microscopy = SPDM). The cells were exposed to ionizing radiation of different doses and aliquots were fixed after different repair times for SPDM imaging. In addition to the repair dependent histone protein pattern, the positioning of antibodies specific for heterochromatin and euchromatin was separately recorded by SPDM. The present paper aims to provide a quantitative description of structural changes of chromatin after irradiation and during repair. It introduces a novel approach to analyse SPDM images by means of statistical physics and graph theory. The method is based on the calculation of the radial distribution functions as well as edge length distributions for graphs defined by a triangulation of the marker positions. The obtained results show that through the cell nucleus the different chromatin re-arrangements as detected by the fluorescent nucleosomal pattern average themselves. In contrast heterochromatic regions alone indicate a relaxation after radiation exposure and re-condensation during repair whereas euchromatin seemed to be unaffected or behave contrarily. SPDM in combination with the analysis techniques applied allows the systematic elucidation of chromatin re-arrangements after irradiation and during repair, if selected sub-regions of nuclei are investigated.

## Introduction

A major goal of ongoing research in biophysics is to understand the potential interaction of nuclear architecture (“structuromics”) and nuclear functions (“genomics”) in order to elucidate the mechanistic principles behind spatial organisation of chromatin and chromatin re-arrangements during intra-nuclear processes like DNA repair after exposure of ionizing radiation (“radiomics”) [1,2]. It is well known that individual chromosomes occupy distinct sub-volumes of a cell nucleus called chromosome territories [3,4,5]. These territories are sub-divided into domains of different compaction levels correlated to functional activities [6,7, 8,9]. One example of such domains is the distinction between decondensed, transcriptionally active euchromatin and tightly compacted heterochromatin generally to be assumed to be inactive [10, 11,12,13,14]. Gene-rich regions tend to be located towards the nuclear interior whereas gene-poor regions are generally found towards the periphery [15,16,17,18]. Up-regulation of genes during tumour genesis or DNA radiation damage response as well as DNA double strand break repair mechanisms were shown to be associated with re-organisation of chromosome territories [19, 20,21,22,23,24,25]. Chromatin on the nano-scale seems to have a dynamic structure [1,26] that allows re-arrangements with high flexibility in order to ensure appropriate functioning or damage response. Chromatin architecture appears to have an influence on the sensitivity to DNA radiation damage [27] and consequently the following repair behaviour [28,29]. Thus, chromatin arrangement reflects genome activities [30] and may correlate to radiation caused damage. Repair processes on the other hand should show systematic re-arrangements due to heterochromatin de-compaction for repair [26,27,31] or sub-diffusive movement of chromatin break ends [32].

In conclusion, many investigations have shown that the detailed study of the genome architecture would offer parameters directly correlated to the damaging process during irradiation and the following repair. This, however, requires global insights in conformation changes of chromatin and appropriate methods of quantification.

Recent technological advances allow the study of the nucleosome positions [histone proteins] in the 3D space and the chromatin arrangement not only on the micro- but also on the nano-scale [33,1] indicating that, besides the organisation of chromosome territories, the chromatin conformation below dimensions of 100 nm, independently of the cell type, is also not random. In order to obtain nanoscopic insights into 3D-conserved intact cell nuclei, light microscopic techniques are required that surpass the diffraction dependent resolution limit described by Abbe and Rayleigh more than hundred years ago [34,35]. In modern microscopes with high numerical aperture objective lenses, this resolution limit is still valid and is about 200 nm laterally and 600 nm axially.

Novel approaches in light microscopy circumventing the Abbe-Rayleigh boundary conditions enable effective optical resolutions down to the order of 10 nm or even better [36,37,38]. One of these techniques is localization microscopy [39,40] which is based on the fundamental concept of optical isolation of objects by different spectral signatures, e.g. by using constant differences in the absorption/emission spectrum (see for instance SPDM [41]); or by using differences in the time domain, for example fluorescence life times [42], or fluorophores/fluorescent proteins that can be switched between two different spectral states (see for instance PALM [43], (F)PALM [44], STORM [45], SPDM [46,47], GSDIM [48] etc.) to achieve a temporal isolation and thus a spatial separation of single signals. This allows the determination of object positions and their spatial distances even if they are very close together, i.e., closer than the Abbe-Rayleigh resolution limit. All acquired positions of fluorescent molecules can be merged into one “pointillist”, super-resolution image, in which the effective resolution is only depending on the localization accuracy and the density of the detected molecule positions.

A certain embodiment of localization microscopy used in the experiments described here is SPDM (Spectral Position Determination Microscopy; [49,46,47]) which has the advantage of using conventional fluorescent proteins or fluorescent fluorophores which are switched to a “dark” state by laser light-induced reversible photo bleaching [50,51]. From this dark state the fluorescent molecules randomly fall back into the emission state and emit their photons when irradiated. Each of the emitting fluorophores is represented by an Airy disc in the microscopic image. The centre-of-mass [barycentre] of such a disc approximates the location of the emitting molecule with high accuracy. So far, the resolution power of SPDM has been demonstrated in 2D-analyses of different applications, e.g. analyses of chromatin in cell nuclei, of proteins in cytoplasm and membranes, of antibody arrangements on cell surfaces and in cell nuclei, etc. [33,39,1,52,53,54].

Although in cellular specimens the typical localization precision and the point-to-point distance resolution of SPDM images presently is in the order of 10–20 nm, pointillist images locally differing in point densities are not obvious to be interpreted considering the complete structural information [53]. Visualization techniques have been developed considering localization accuracy and point density [55,56]. Images showing solely the detected molecule positions (or the positions blurred with the individual localization accuracy) give a better idea of the scattering of the detected points but make it harder to recognise the (correct) underlying structures by the human visual system especially if they represent the result of a dynamic process like chromatin re-arrangements during DNA damage repair after ionizing radiation exposure.

Therefore, we present novel evaluation procedures of SPDM images based on statistical physics and graph theory to provide a quantitative description of structural changes of chromatin after ^137^Cs irradiation and during the following repair processes. Histones labelled with fluorescent proteins by transfection [56] or fluorescent antibodies specific for heterochromatin or euchromatin mark the point positions of individual histone proteins or individual labelling sites in heterochromatic or euchromatic regions, respectively, and are detected by SPDM in a 2D optical section through the cell nucleus. Statistical analyses of specimens exposed to different doses and fixed at different time points post irradiation will allow the investigation of the chromatin conformation changes during the repair process.

## Materials and Methods

### Cell culture and irradiation

In all experiments stably transfected HeLa cell lines, originally developed by Tobias A. Knoch [57], expressing either green fluorescent protein (GFP) labelled histone H2B or yellow fluorescent protein (YFP) labelled histone H2A [58,59], were cultured in an incubator at 37°C and 5% CO_2_ according to standard cell culture protocols. The used RPMI 1640 medium (Life technologies GmbH, Darmstadt, Germany) was composed of 10% fetal bovine serum (FBS) (Biochrom AG, Berlin, Germany), 1% penicillin (stock: 5000 units/ml) / streptomycin (stock: 5000 μg/ml) (Life technologies GmbH, Darmstadt, Germany) and 1% L-glutamine (stock: 200 mM) (Life technologies GmbH, Darmstadt, Germany). Additionally 10 mM N-2-hydroxyethylpiperazine-N-2-ethane sulfonic acid (HEPES) (Carl Roth GmbH, Karlsruhe, Germany) was added to provide an extra buffering capacity for cells which has an extended period outside the C0_2_ incubator (e.g. irradiation process). The cells were passaged in T75 culture flasks over 2–3 days with a dilution of 1:10. For each radiation experiments approx. 75.000 cells were seeded in Lab-Tek Flaskette Slide/Flask (VWR International GmbH, Darmstadt, Germany) and incubated for 2 days (until 70%–80% confluency).

Afterwards the cells were irradiated with γ-rays of two opposing ^137^Cs sources. The dose rate of both sources was 0.5 Gy / min. The cells received a total dose of 0.5 Gy, 2 Gy or 3.5 Gy / 4 Gy. From each of these samples aliquots were fixed with 4% formaldehyde 30 minutes, respectively up to 48 hours after irradiation. In addition non-irradiated specimens were prepared as control (see also [33]). Samples prepared for the study of the nucleosomal positions were not immunostained.

### Immunostaining of heterochromatic and euchromatic regions

Immunostaining of heterochromatic and euchromatic regions was performed according to a standard protocol on separate cell samples. The cells were permeabilized with 0,2% Triton X-100 (Merck KGaA, Darmstadt, Germany) diluted in 1 x PBS (Life technologies GmbH, Darmstadt, Germany). After blocking with 2% bovine serum albumin (BSA) (Carl Roth GmbH, Karlsruhe, Germany) in 1x PBS the antibodies were diluted in 2% BSA blocking solution. The used primary antibodies were either directed against H3K4me3 (= mainly euchromatin regions) or H4K20me3 (= mainly constitutive heterochromatin regions) (Abcam, Cambridge, Great Britain). Secondary antibodies (Life technologies GmbH, Darmstadt, Germany) labelled with Alexa568 (λ_ex/em_ = 560 nm / 610 nm) were used for the fluorescence imaging. All samples were counterstained using DAPI (λ_ex/em_ = 364 nm / 454nm) and embedded in Prolong Gold (Life technologies GmbH, Darmstadt, Germany) before SPDM imaging.

### Localizaton microscopy

The following parameters refer to the SPDM setup. For the excitation of the fluorescent proteins GFP (green) or YFP (yellow), a laser with an excitation wavelength of 488 nm was used (Coherent (Deutschland) GmbH, Dieburg, Germany). The antibody fluorescence of Alexa568 was excited by a laser with the wavelength of 568 nm (Coherent (Deutschland) GmbH, Dieburg, Germany). The laser intensity was about 13 kW / cm^2^ (488nm) and 25 kW / cm^2^ (568nm). For each SPDM image further evaluated, a time stack of about 1000 images was acquired with an integration time of 55 ms (GFP/YFP) or 150 ms (Alexa568). The detection-off time between single images of these time stacks was 1 ms.

Optical isolation of signals was achieved utilizing a light-induced reversibly bleached state of GFP, YFP or Alexa568. By illuminating the sample some fluorescent proteins or dye molecules are bleached irreversibly while another amount is transferred to a reversibly bleached state. The stochastic recovery of these molecules to the fluorescent state can be used for optical isolation of the detected single molecule signals.

The laser beam was expanded by a factor of 2.5 before being focused into the back focal plane of an oil immersion objective lens (HCX PL APO, 63×, NA = 0.7–1.4, Leica, Wetzlar, Germany). The emitted fluorescent light passed a dichroic mirror (AHF Analysetechnik, Tübingen, Germany) and an appropriate blocking filter before being focused onto the charge-coupled device camera (SensiCam QE, PCO Imaging, Kehlheim, Germany). An additional lens was mounted in the excitation pathway for increasing the laser intensity in the object plane to obtain appropriate conditions for the reversible photo bleaching (for details see [49]).

### Segmentation of Images

In order to detect the regions of interest, a segmentation of the image is performed in the first step of the analysis. In this process the areas that have a considerably lower than average number of localized fluorophores are excluded. This includes the area that is on the outside of the cell nucleus but also areas inside the nucleus, e.g. at the site of nucleoli.

In the image, the fluorophores represent a set of points in the two-dimensional plane. To perform the segmentation a density distribution for the fluorophores on the entire image was calculated by applying a Gaussian kernel density estimation [60]. This means that each point on the image is replaced with a two-dimensional Gaussian probability distribution with a mean value corresponding to the coordinates of the point. Radially symmetric Gaussian distributions are chosen by setting the bandwidth matrix to a multiple of the unit matrix. The multiplier is a parameter in the model which defines a correspondence between the standard normal distribution and the physical scales in the experiment, and it is set to 40 nm. The sum of all the Gaussian probability distributions is then renormalized yielding a probability distribution for the points of the image. Since the values of the probability distribution are proportional to the density of points, the described procedure yields an accurate density distribution of the points on the complete image.

Using the density distribution, a mask is calculated that accepts all areas with a density that is above 25% relative to the lowest value, and blanks out all other areas. In order to make sure that also the low density regions at the border of the cell nucleus are excluded from the analysis, the boundary of the masked areas are further reduced by another 150 nm. Thus areas that are outside of the cell nucleus and areas which have a low marker density (e.g. sites of nucleoli) are efficiently excluded.

### Radial Distribution Function

The radial pair correlation function *g*(**r**_**1**_,**r**_**2**_)) is a measure of structure for many particle systems such as liquids. It is the probability distribution function that two particles are at the positions **r**_**1**_ and **r**_**2**_. Since absolute positions are not important, the correlation function is in fact only dependent on the directed distance between the two positions **r** = **r**_**12**_ = **r**_**2**_ − **r**_**1**_. Thus the radial distribution function can be simply written as *g*(**r**) and is given by

$$g(r)=\frac{1}{N}\langle \sum_{i=1}^{N}\sum_{j=1}^{N}\delta (r-r_{\mathrm{ij}})\rangle$$

The probability to find a second particle in the directed distance **r** of the first particle is *g*(**r**)*d* **r**. Assuming an isotropic system, the pair correlation function only depends on the undirected distance *r* = |**r**|, and the probability to find a second particle in the distance *r* of the first particle is given by 4π*g*(*r*)*dr*.

For each image the pair correlation function is determined by calculating all pair distances between points inside of the masked regions of interest. Due to the finite system size, not all points can be used for the calculation of *g*(*r*). Points that are close to the border of the masked image have a different surrounding than the ones in the bulk, therefore including these points in the calculation of *g*(*r*) would distort the results. To take this into account, first the masked image was isotropically reduced from the border by a distance *d*_*shrink*_, thus yielding a second masked image that is considerably smaller than the first one. All points in the second mask have then equal environments for distances up to *d*_*shrink*_. Then, for each of the points in the second mask the distances to all other points that lie in the original mask were calculated. The normalized distribution of these distances then yields the radial distribution function *g*(*r*) for the image.

To observe the generic behaviour, the radial distributions obtained for the different images were averaged. The standard deviation of the mean value is then used as a measure for the uncertainty of the averaged *g*(*r*) at each distance *r*.

### Analysis by Graph Topological Approach

Another possibility to gain insight in the structure presented by the localization data is to build a graph on the skeleton defined by the localized points. This graph then can be investigated by means of graph theoretical methods and the topological or relational properties of the underlying structure can be concluded.

Graphs [61] are abstract mathematical objects designed to capture interdependencies of certain entities. Entities are represented by nodes (or vertices) while the connections are encoded by edges between nodes. A graph is usually denoted by *G*(*E*,*V*), where *V* is the set of nodes and *E* = {(*a*,*b*)|*a* ∈ *V*,*b* ∈ *V*} is the set of edges. Graphs have been used to study a variety of structures and phenomena [62, 63, 64, 65, 66], most of these studies relying on the mathematical field called graph-theory.

In the present study, the main interest is the observation of local properties of the geometric arrangements formed by the points. Therefore, the chosen graph building procedure must construct graphs that emphasize the local relations among the fluorophores. In other words, a nearest-neighbour graph (NNG) must be built, in which each node is connected with its first-order neighbours.

**Fig 1:**
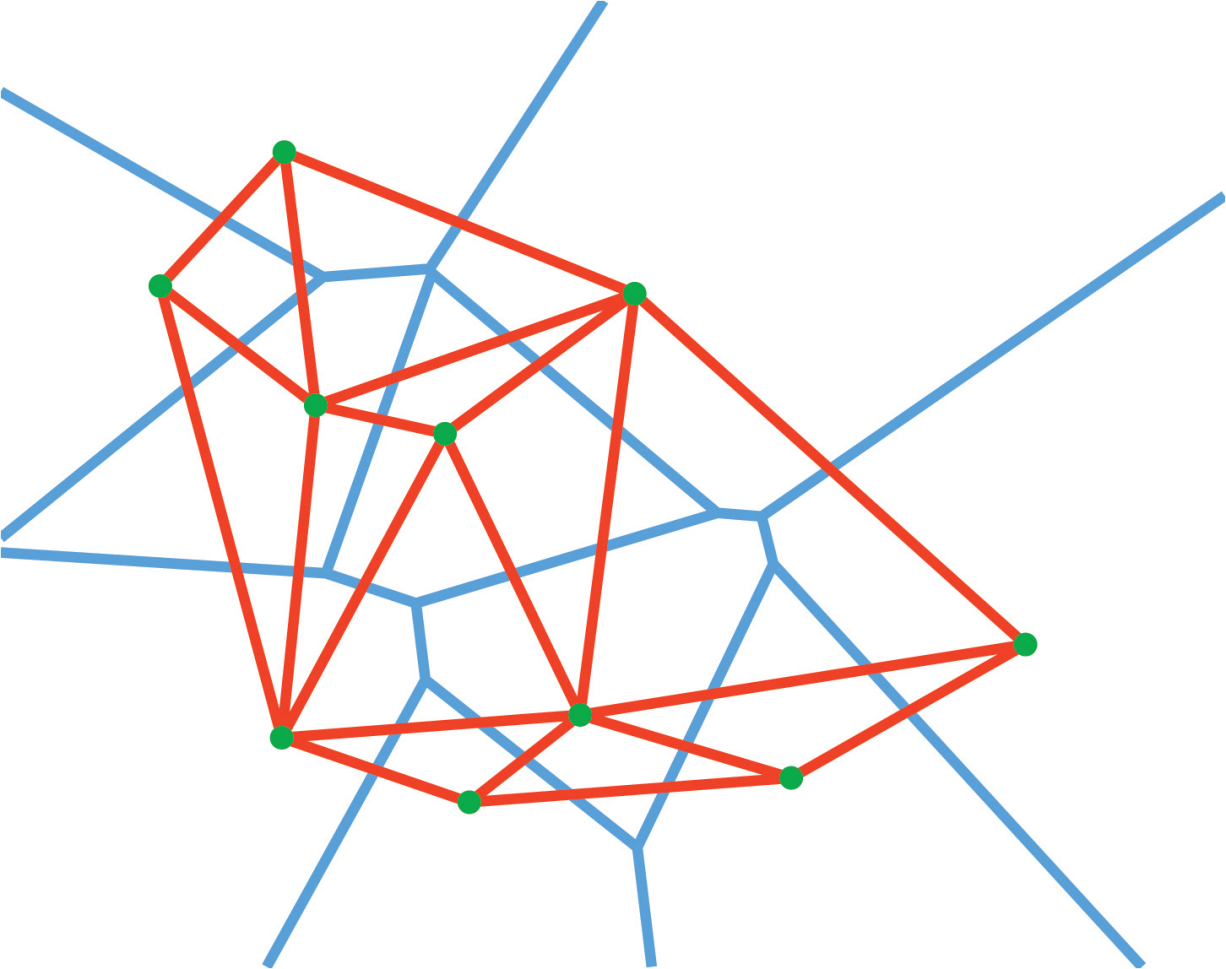
Sketch of a Delaunay Triangulation. The Delaunay triangulation and its dual, the Voronoi tessellation for a random set of points. The blue lines are the segments of the Voronoi tessellation, the red ones are the edges of the Delaunay graph (triangulation).

There are many possible ways to build the NNG for a given set of points. The most widely used approach is the construction of the Delaunay triangulation [67]. In order to have an intuitive understanding of this method, let us first introduce the dual of this triangulation, the Voronoi tessellation [68]. By definition, the Voronoi tessellation of a set of points *S* is a tessellation in which each Voronoi cell *V*_*i*_ corresponding to the site *S*_*i*_ consists of all the points of the space closer to *S*_*i*_ than any other site. The faces of the Voronoi diagram consist of all the points in the space that are equidistant to sites corresponding to touching Voronoi cells. Thus the Voronoi cell *V*_*i*_ defines the space dominated by the site *S*_*i*_. The Voronoi tessellation can be transformed into the Delaunay triangulation by connecting the sites of the neighbouring Voronoi cells. Two points will be considered neighbours if they are directly connected by an edge of the Delaunay triangulation or equivalently, if their corresponding Voronoi cells are touching. Fig 1 illustrates the Voronoi tessellation and the Delaunay triangulation for a given set of points. Through the Delaunay triangulation, the NNG is constructed for each experiment and two properties of the obtained graphs are calculated: the rescaled probability density *f*(*r*) of the edge lengths and the conditional probability *p*(*r*|*r*′) of the edge lengths.

Let us denote the probability distribution of the length of the edges in a given graph *G*(*E*,*V*) obtained by the Delaunay triangulation of a point-set corresponding to one of the localization images by $\overset{-}{f}(r).$ For different experiments different $\overset{-}{f}(r)$ distributions are expected. The differences may stem either from different underlying structures or from different experimental conditions as these can vary from experiment to experiment (slightly different concentration of stains or different microscope gain). Provided that different experimental conditions have a liner effect on the density *ρ* of marked sites, variations $\overset{-}{f}(r)$ can be eliminated. This is achieved by rescaling the distributions to a reference density *ρ*_0_. For this, the following procedure is applied: Since the $\overset{-}{f}(r)$ functions are probability distributions, the edge lengths, in fact, are treated in a probabilistic manner, that is, *r* is considered a random variable.

Thus, the rescaling of the distributions to a reference density *ρ*_0_ is in fact a probability transformation over *r*. To see this, let *d*_*ij*_ = *d*[(*x*_*i*_,*y*_*i*_),(*x*_*j*_,*y*_*j*_)] denote the length of the edge between vertexes *i* and *j*. If the coordinates (*x*,*y*) of all the points are multiplied with a positive real number *α* = *ρ*_0_/*ρ* the following relation holds:

$$d[\left({\alpha x}_{i},{\alpha y}_{i}),({\alpha x}_{j},{\alpha y}_{j}\right)]=\alpha d[\left(x_{i},y_{i}),(x_{j},y_{j}\right)].$$

On the other hand, this multiplication corresponds to a uniform dilation (or contraction) of the system, that is, a uniform scaling of the density. Therefore, to calculate the scaled probability density, a probability transformation defined by

t(r)=αr

must be applied. Using basic probability theory, the rescaled probability density

$$f(r)=\frac{1}{\alpha }\overset{-}{f}(\frac{1}{\alpha }r)$$

is obtained.

The reference density *ρ*_0_ can be, for instance, arbitrarily defined and the corresponding transformations for the different experiments can be carried out. Alternatively, the probability densities can be fit to each other, using *α* as a fitting parameter.

Certain local properties are averaged out both by the *g*(*r*) and the *f*(*r*). For instance, non-regular density variations are not captured by these measures. Another example is a situation when certain regular structures appears multiple times but the size of the structures varies.

**Fig 2:**
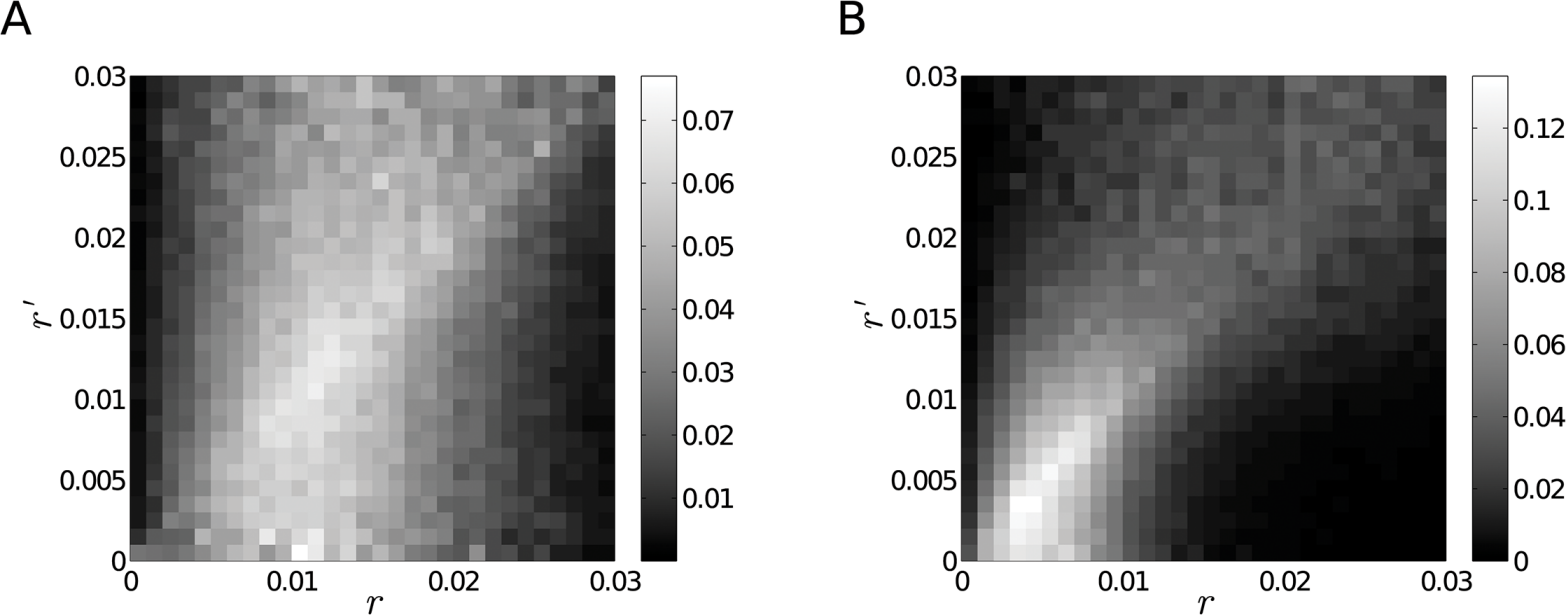
Conditional Probabilities of Edge Lengths for Random Data. The figures illustrate how the *p*(*r*|*r*′) conditional probability looks for randomly generated point positions. Panel A. Conditional probability of points with coordinates generated according to a uniform distribution. Panel B. Conditional probability of points with coordinates generated according to a mixture of uniform distribution and clusters of Gaussian distributions. In the latter example an emphasized diagonal is observed which is the result of the Gaussian clusters. Tightly packed points tend to produce short edges, while points from the edges of the clusters mostly have longer edges.

In order to detect these situations, the conditional probability *p*(*r*|*r*′) of the edge lengths of the nearest neighbour graph is calculated. This conditional probability is the probability of finding an edge with length *r* attached to a node which for sure has an edge with length *r*′. The conditional probability can numerically be represented in a matrix structure *P* where each row corresponds to a condition for a given interval over *r*′ and each column represents an interval over *r*. The matrix entry *P*_*ij*_ corresponding to a given row *i* and a column *j* will give the probability of finding an edge with a length between *r*_*j*_ and *r*_*j*_ + *dr* attached to a node which has at least one edge with a length between *r*_*i*_′ and $r_{i}^{\prime }+dr$. In case the localization points are arranged according to a specific structure, the *P* matrix will indicate a preferential attachment. For instance, in a long chain which has constant link-lengths over larger domains, but different domains have different link lengths, the *P* matrix will be almost diagonal. Fig 2 shows the *P* matrix for two randomly generated data-sets, one set containing points with coordinates distributed uniformly while the other set has additional Gaussian clusters. As the figure illustrates, uniformly distributed points will produce a rather uniform matrix, while point-sets with clusters will have a more emphasised diagonal.

## Results

Stably transfected HeLa cells expressing either GFP labelled histone H2B or YFP labelled histone H2A were fixed and embedded in antifade media for SPDM imaging. The cell nuclei were selected before SPDM imaging by visual inspection of wide-field images. A focus section of a depth of about 600 nm was chosen through the centre of each nucleus. About 1000 images of the same section were acquired and merged. The stochastic blinking of the fluorescent proteins was registered during the image stack and resulted in a localization map for all molecules detected. Typically about 70,000 fluorescent points were registered in the given optical section of a nucleus.

### Exposure to ionizing radiation cause local but no global changes of the genomic organisation

**Fig 3:**
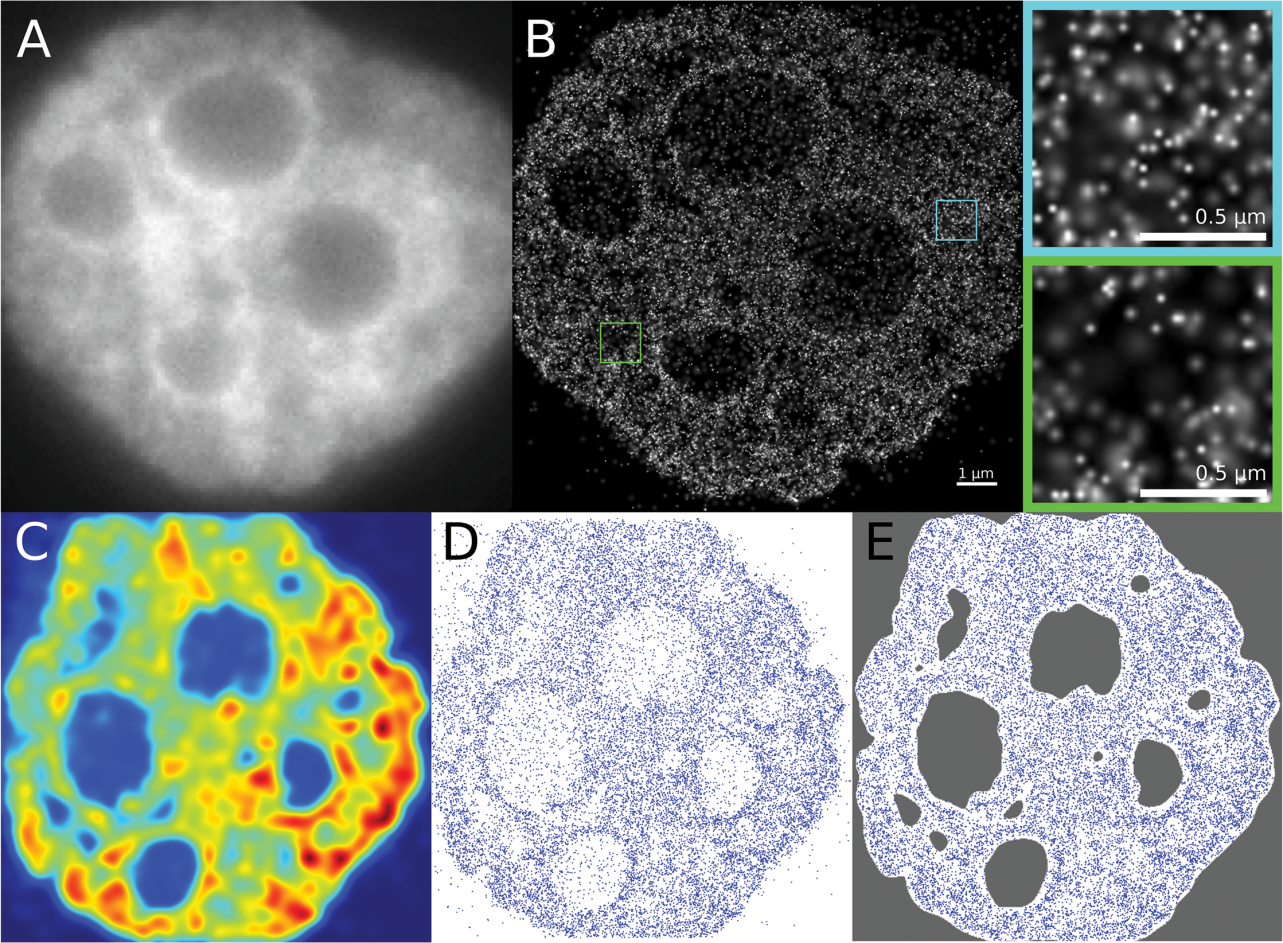
Localization Microscopy Images. Panel A. Widefield image of a cell nucleus, as usually obtained by standard microscopy. Panel B. Pointilist image obtained after merging the acquired time series of SPDM images. Each point represents a detected blinking process during image acquisition. The green insert shows an area with a low average density, which could correspond to euchromatin and the blue area corresponds to an area with high point density, possibly belonging to heterochromatin. Panel C. This panel shows the calculated density distribution of the localized markers using a Gaussian kernel density estimation with a uniform Gaussian kernels. Panel D. The figure shows the localized points of the image. From the points, areas with very low point density, possibly corresponding to nucleoli, can clearly be made out visually. Panel E. Shown is the segmented image where only the area of interest is kept for the analysis. The segmentation was based on the density distribution; areas below a threshold density were discarded for analysis.

From the experimental performance it can be assumed that the radiation damage sites were distributed all over the whole cell nucleus. Since they were not labelled specifically, the first approach was to globally study the distribution of nucleosomes represented by the blinking fluorescent proteins and their spatial variations after irradiation and during repair. The following processes describe the image analysis procedures:

At first a segmentation of each of the images is performed to cut out those areas in the image that do not belong to the cell nucleus. Additionally, areas inside the nucleus that have a very low density of markers are neglected since these areas are indicative for nucleoli. The segmentation is based on the calculation of the density distribution of markers in the image. Only the areas with a density of more than 25% of the largest density are taken into consideration. Fig 3 shows an example of an image with the localized H2B histones and their density distribution and the final segmented image in comparison to a widefield image as usually obtained by standard microscopy. To assess how the overall organization of chromatin changes induced by ionizing radiation compared to non-irradiated cells, the pair correlation function *g*(*r*) for localized H2B histones in non-irradiated cells and cells that were exposed to ionizing radiation is calculated. The pair correlation function is a measure for the positional correlation of points in a many-particle system. At this, 2π*rg*(*r*)*dr* denotes the probability of finding two points separated by a distance between *r* and *r* + *dr* for infinitesimal *dr*.

**Fig 4:**
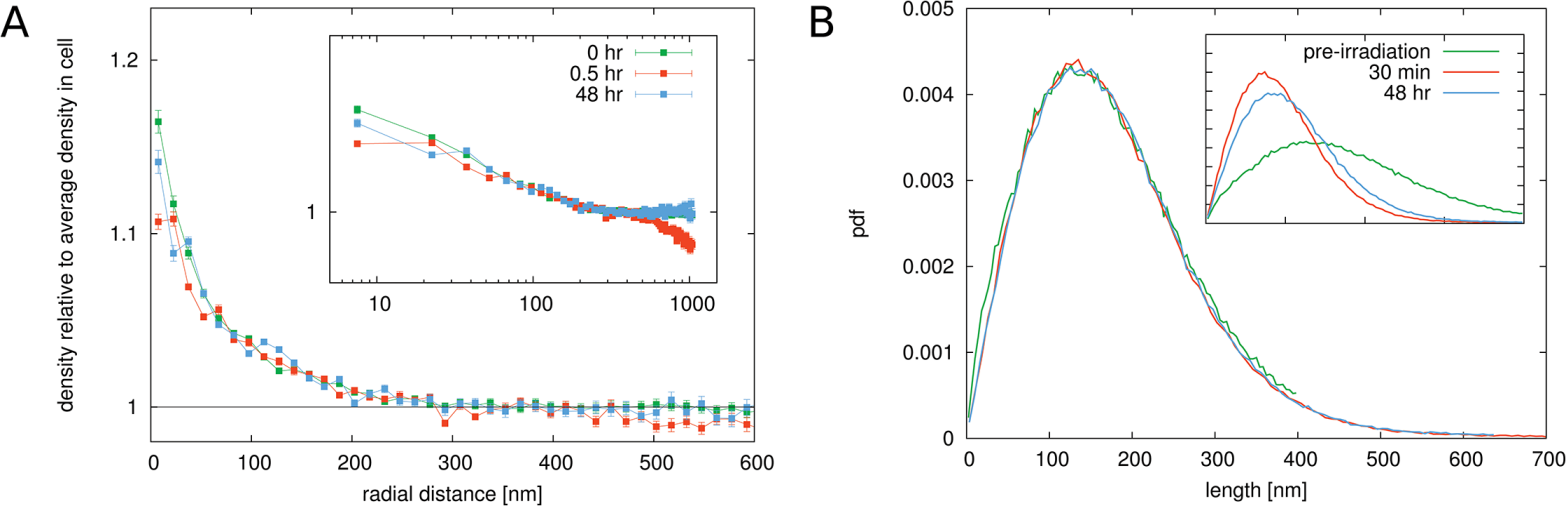
Results for cells exposed to 0.5 Gy radiation. Panel A. Radial pair correlation functions determined at different repair times after exposure to 0.5 Gy radiation dose. The radial pair correlation function *g*(*r*) shows that correlations between the positions of labeled H2B histones exist up to a distance of roughly 300 nm. Marker and thus chromatin densities in the surroundings of each marker are elevated compared to the average density of markers in the cell nucleus. Above 300 nm, however, histone positions are uncorrelated and the marked histones can be viewed as being positioned randomly relative to each other. Furthermore, the radial pair correlation function for cells exposed to 0.5 Gy γ-irradiation apparently does not differ from the one of untreated cells, regardless of the time passed after irradiation. Panel B. Rescaled distribution of the length of edges in a Delaunay triangulation of the H2B marker positions after different repair times post 0.5 Gy irradiation. The inset shows the original distributions for the different images. The differences in the edge length distributions are due to the different marker densities in the images. Therefore a rescaling of the distributions was performed with respect to the point density to clear out this effect. The rescaled distributions can be seen in the main panel and show that the distributions belong to the same family.

Typical results for the radial pair correlation function of H2B markers in non-irradiated and irradiated cells are shown in Fig 4 panel A. Images of non-irradiated cells and cells fixed 30 minutes respectively 48 hours after exposing them to 0.5 Gy, 2 Gy and 4 Gy of γ-irradiation were analysed. For all setups a deviations of *g*(*r*) from unity only for distances up to 300 nm were detectable. It thus shows that structured organisation of chromatin in the cell nucleus is only apparent up to distances of roughly 300 nm. Below this critical distance, locations of labelled histones H2B are visibly correlated. However, on distances larger than 300 nm the radial correlation function drops to unity and the locations of histones can be viewed as being randomly distributed relative to each other. The radial distribution functions for the H2B histones are lying on top of each other and no deviations can be seen for cells that were exposed to ionizing radiation. Since static chromatin architecture is in contrast to all findings so far, it seemed to be obvious that such results could be interpreted as the result of averaging distance distributions over the complete cell nucleus.

In order to assess the local positional correlations that are apparent up to a length of 300 nm a graph theoretical approach was pursued. First of all, the nearest neighbour distance distribution of the localized fluorophores was calculated. For this, a Delaunay triangulation was performed to obtain graphs for all points in the segmented images and then the length distribution of the edges of the graph was calculated. Results for H2B markers for non-irradiated cells and cells fixed 30 minutes and 48 hours, respectively, after exposure to ionizing radiation are shown in Fig 4B. A significant difference between them can be observed (see inset). However, the distributions belong to the same family since the rescaled distributions *f*(*r*) are exactly the same. Therefore we can state that the observed differences may stem from different experimental conditions such as different overall recorded marker densities. This example demonstrates that it is necessary for comparison to rescale detected images to a comparable amount of points since labelling efficiency as well as detection efficiency of the microscope may vary between different series of experiments.

**Fig 5:**
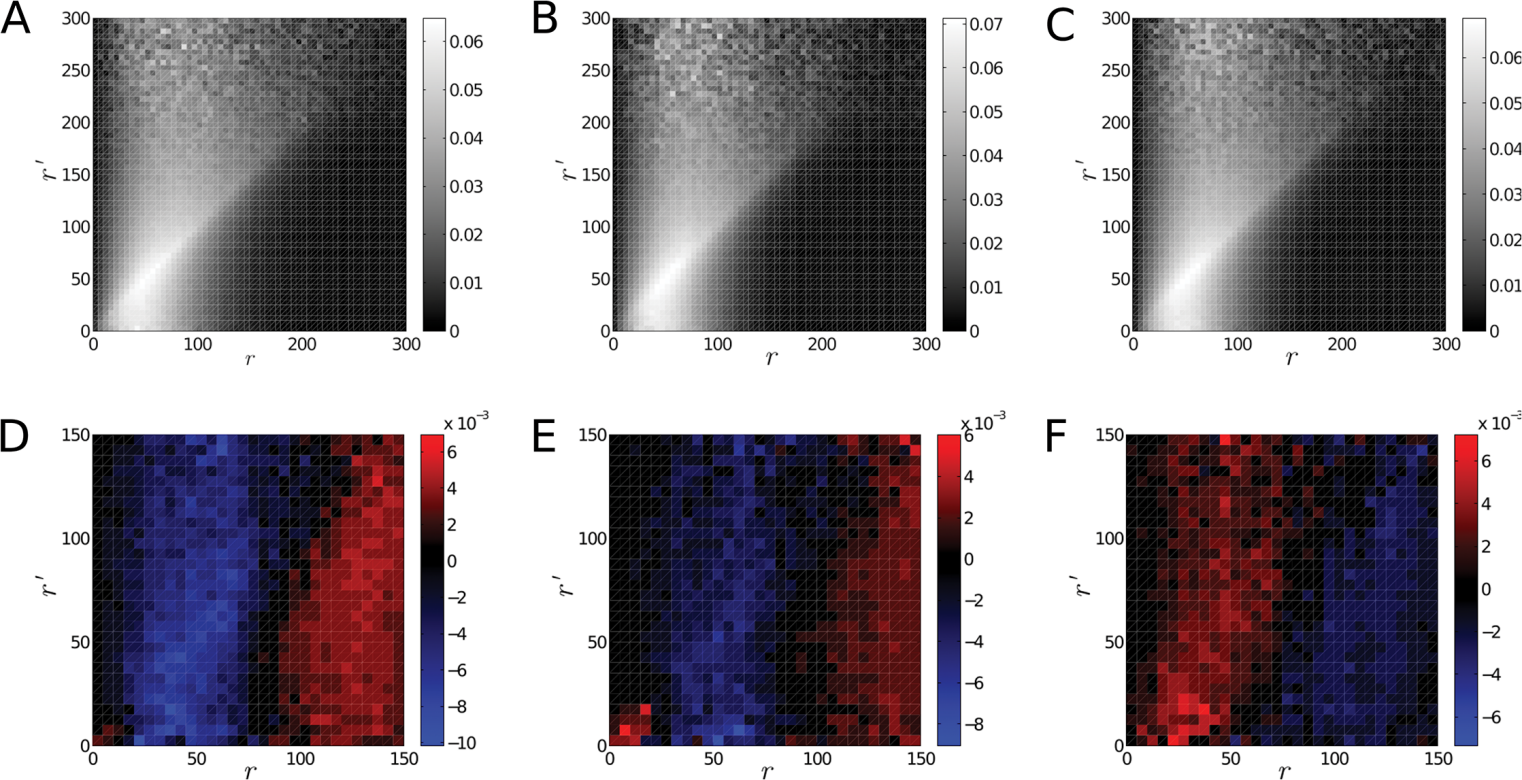
Conditional Probability Distribution of the Edge Lengths. Panel A. The panel shows the conditional probability distribution *p*(*r*|*r*′) of the edge lengths for the H2B markers before irradiation. The relatively prominent diagonal indicates locally a varying density. Panel B. The panel shows the conditional probability for the H2B markers 30 minutes after irradiation. Panel C. The panel shows the conditional probability for the H2B markers 48 h after irradiation. Panel D. The panel shows the difference of the conditional probabilities *p*(*r*|*r*′) measured for structures recorded before irradiation and for structures registered 30 min after irradiation. Shades towards red indicate values which are larger in the samples before irradiation, while values towards the shades of blue indicate probabilities which are larger in images registered 30 min after irradiation. The plot indicates slightly increased values along the diagonal after irradiation. This might mean a slightly increased clustering of the points. Panel E. The panel shows the differences of the conditional probability before irradiation and 48 h after irradiation. Entries in shades of red are larger in samples recorded before irradiation, while entries in shades of blue are larger in the samples recorded 48 h after irradiation. The trend is similar to that observed in panel D, however differences are less prominent. Panel F. The panel shows the differences if the conditional probability measured 30 min and 48 h after irradiation respectively. The red shades indicate larger probabilities in samples recorded 30 min after irradiation while blue shades indicate larger probabilities in samples registered 48 h after irradiation. Here a reversed trend compared to panel D was observed.

Additionally, a calculation of the *p*(*r*|*r*′) conditional probability for the localized H2B markers was performed. The matrix representations *P* of the conditional probabilities are plotted in Fig 5, the three panels A, B and C corresponding to pre-irradiation configuration and to configurations fixed 30 minutes and 48 hours post-irradiation, respectively. While the plots resemble a mixture of the plots from Fig 2, they do not exhibit obvious big discrepancies amongst each other. Panels D, E and F in Fig 5 present the differences between the three panels A, B and C. These plots show that for samples imaged shortly after irradiation, the diagonal of the conditional probability is more prominent but for samples recorded after a longer healing time the diagonal recedes. This means that, while upon irradiation the system appears to change towards a less uniform structure, that is towards a less homogeneous *P* matrix, with longer healing time the changes are reverted. These results indicate that after irradiation a formation of nucleosome clusters seem to increase whereas in the later repair phase this effect decreases.

In all cases the results of YFP-labelled H2A did not differ from GFP-labelled H2B (data not shown).

### Heterochromatic regions show a decondensation upon exposure to γ-radiation

**Fig 6:**
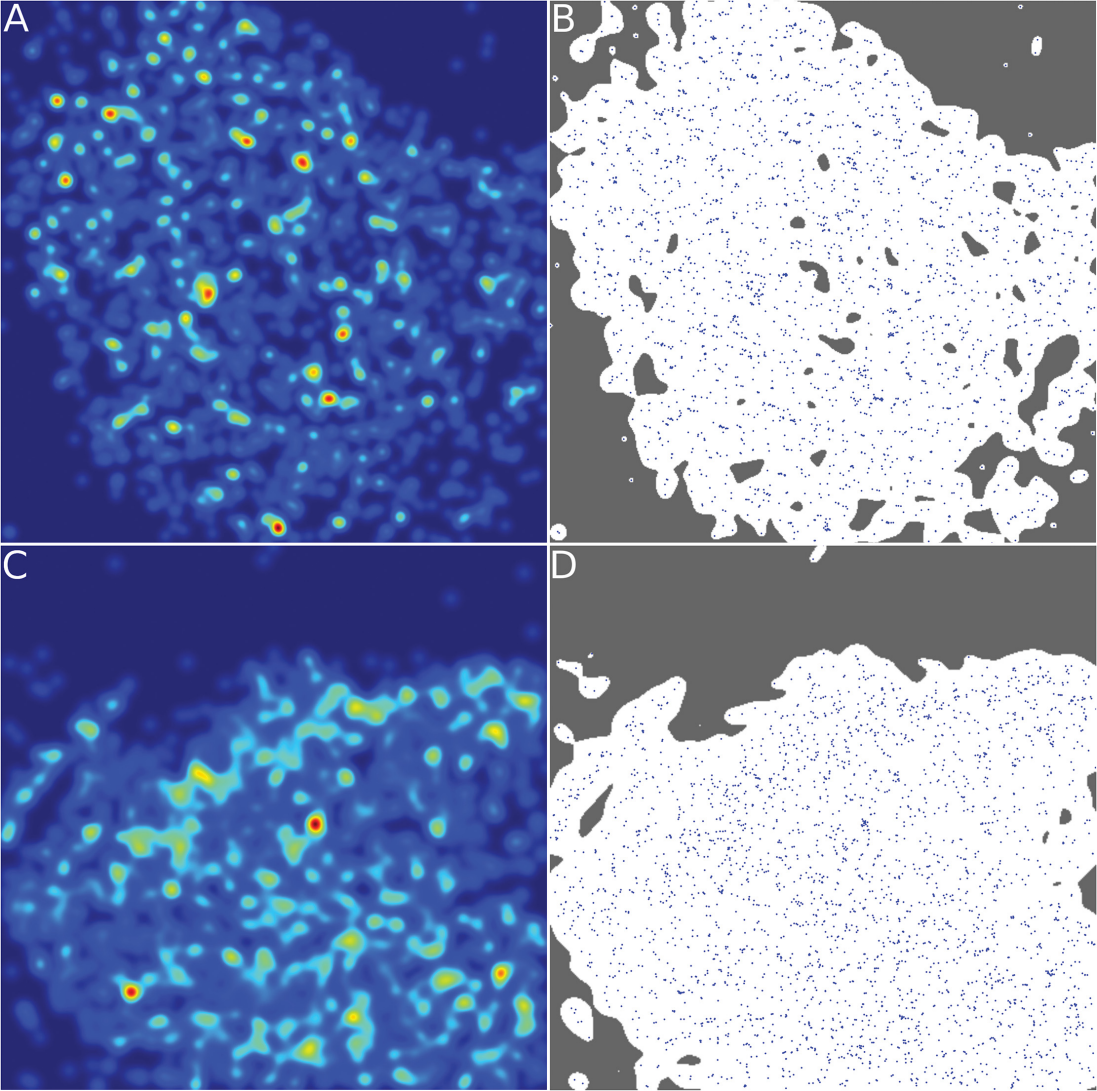
Examples of Localization Microscopy Images of Heterochromatin Markers. Panel A. Shown is the density distribution of the localized markers in a cell prior to irradiation. Small, very bright spots where markers are agglomerated can be seen. This means that heterochromatin is mainly organized in coarse areas. Panel B. Shown is the segmented image of the not irradiated cell that is used for subsequent analysis of the marker distribution. Panel C. The density distribution of a cell at 30 min after irradiation with 0.5 Gy is shown here. Differences between this cell and the not irradiated cell can be made out by visual inspection. We observe that the density has much less agglomerated and bright spots and is instead much more homogeneous. Panel D. This effect can also be seen by visual inspection of the heterochromatin markers directly. Marker positions are visibly more spread out and less strongly clustered together. Heterochromatin clearly undergoes structural changes upon irradiation.

In addition to the stably nucleosome labelling by fluorescent proteins, antibodies for H4K20me3 were used as markers for heterochromatic regions. These experiments were carried out independently from the ones that focus on the positions of the nucleosomes, and a separate set of cells was prepared and immunolabeled according to the procedure introduced in the Methods section. Images were processed and segmented with the same methods described above and in the Methods section. In Fig 6 the density distribution of the heterochromatin antibody markers on panel A and the segmented image on panel B is shown. The density distribution shows clearly how heterochromatic regions can be seen as coarse structures located within the nucleus. These areas are visible as bright spots in the density distribution. All localized H4K20me3 antibodies, which were not discharged by the segmentation step, were considered in the calculations. That is, the presented results characterize post-irradiation heterochromatic regions on average, and not only domains where double-strand breaks occurred.

**Fig 7:**
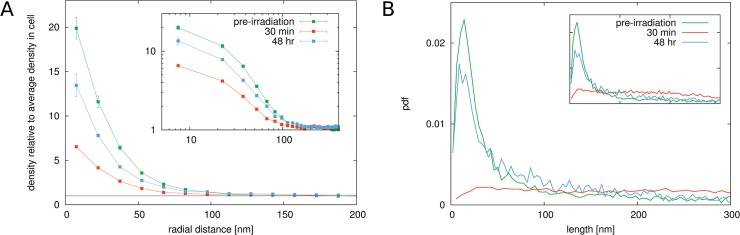
Results for cells exposed to 0.5 Gy radiation. Panel A. The figure shows the radial radial distribution function for H4K20 antibodies representing heterochromatin (methylated histone variants) for non-irradiated and irradiated cells. Error bars represent the standard deviation of the mean value after averaging over the sample of cells. The value for *g*(*r*) at small distances goes up to around 20, indicating the high marker densities in regions where heterochromatin is located. The rapid drop off of the radial distribution function within a distance of less than 100 nm shows that heterochromatin forms small clustered areas that are spread throughout the cell nucleus. Upon exposure to 0.5 Gy γ-irradiation, a dramatic change in the correlation function can be observed in cells that were imaged after 30 min. The value at small radial distances drops to around 6, or around 70% smaller than in non-irradiated cells. This indicates that the density in the heterochromatic regions becomes on average much lower in irradiated cells. In cells measured 48 h after irradiation, the correlation function have recovered again and the value at small *r* is at around 14, only 30% less than in unirradiated cells. Panel B. The distribution of edge lengths in the Delaunay triangulation of the markers confirms these observations. A sharp peak in the distribution at around 30 nm can be seen in untreated cells. In 30 min post-irradiation cells the peak vanishes and a spread distribution can be seen. In 48 h post-irradiation cells however, the peak reappears again but less pronounced than in untreated cells.

The calculated radial distribution function for localized H4K20me3 antibodies in non-irradiated cells and cells exposed to 0.5 Gy dose after different times are shown in Fig 7A. The correlation function for the heterochromatin antibodies shows apparent differences to the correlation function for H2B histones. The high value of up to 20 times the average density that can be found at small distances *r* reflects the fact that antibodies are in very close proximity of each other, indicating compact heterochromatic domains. The quick decay of *g*(*r*) to the average density in the nucleus indicates that these areas are relatively small confirming the visual impression of the images.

Upon irradiation, the value of the correlation function drops for small distances *r*. This means that the average density of antibodies in the surrounding of each antibody is lower during the first repair phase. It indicates that the overall chromatin density becomes smaller in the heterochromatic areas. This could be verified within the nucleosomal pattern 5 min after radiation exposure if the analysed regions of interest were locally reduced to areas only highlighted by heterochromatic antibody accumulation (data not shown).

It can be concluded that regions of constitutive heterochromatic chromatin on average become less compact, i.e., the strongly compacted organisation opens up and adopts a more loose structure. An average drop of 70% of the mean antibody density in a sphere with a radius of 30 nm around an antibody for cells irradiated with 0.5 Gy γ-irradiation after 30 minutes was observed. Therefore, DNA double-strand breaks caused by irradiation in heterochromatin seems to require a drastic decrease of the chromatin density and a strong relaxation of the compact organisation of the chromatin fibre in order to make the damaged sites accessible for repair. 48 h after irradiation, this value is only at around 30% which indicates that structures seem to have recovered after successful repair of DNA damages.

The conclusions drawn from the radial distribution function are verified by the graph theoretical analysis. In Fig 7B the length distribution of the edges in the Delaunay triangulation of the marked H4K20 antibodies is shown. The distribution for non-irradiated cells shows a very characteristic peak at small distances centred at around 30 nm. This emphasizes that there is a characteristic nearest-neighbour distance for the H4K20 markers thus meaning a preference of them to form compact areas. In cells at 30 min after exposure to ionizing radiation, the sharp peak in the edge length distributions vanishes. Instead, the distribution becomes a uniform distribution up to large lengths. The disappearance of the peak is a clear indicator that the heterochromatic regions are no longer organized as compact areas in the nucleus. This difference can be seen visually in Fig 6C. Compared to the non-irradiated cell nucleus shown in Fig 6A, it can be observed that the very bright spots have mostly vanished and the heterochromatic regions in the irradiated cell are much more smeared out. The graph theoretical analysis therefore verifies the observations of the behaviour of the radial distribution function. The heterochromatic regions undergo a decondensation upon exposure to ionizing radiation. At 48 h after irradiation, the peak in the edge length distribution emerges again. Just as the pair correlation function is again very similar to that of non-irradiated cells, the edge length distribution has also the same shape as in the case of non-irradiated cells.

**Fig 8:**
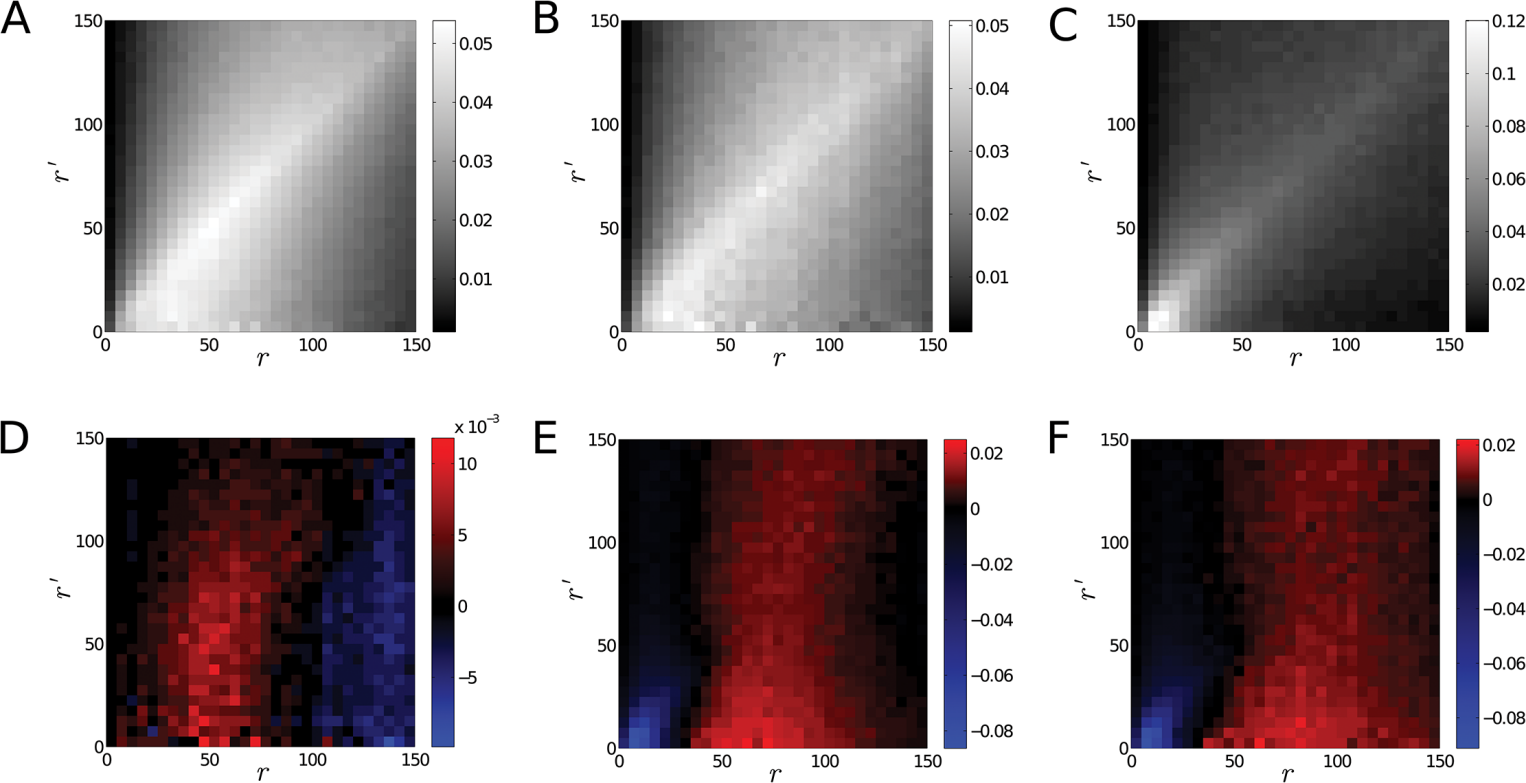
Conditional Probability Distribution of the Edge Lengths for Heterochromatin Markers. Panel A. The panel shows the conditional probability distribution *p*(*r*|*r*′) of the edge lengths calculated for the positions of the antibodies marking heterochromatic regions before irradiation. Panel B. The panel shows the conditional probability for the heterochromatin markers 30 min after irradiation. Panel C. The panel shows the conditional probability for the heterochromatin markers 48 h after irradiation. In all three cases the diagonal is emphasized indicating preferential spatial distribution of the edges. This may stem from the clustering of the heterochromatin markers. Note that although shades along the diagonal are darker in panel C, the value of the corresponding probabilities are very close to the probabilities along the diagonal of panel A except for small radii. Panel D. The panel shows the difference in the conditional probability distribution *p*(*r*|*r*′) before irradiation and 30 min after irradiation. A red shade of the color-map means that the probability is higher before irradiation while a blue shade means that it is higher after irradiation. The panel indicates a slightly stronger change along the diagonal indicating a more homogeneous system after irradiation. However, the change is almost independent of the value of the condition *r*′. Panel E. The panel shows the difference of the conditional probability distribution calculated for samples before and 48 h after irradiation. Shades of red indicate higher probabilities for the samples recorded before irradiation while shades of blue indicate higher probabilities in samples recorded after irradiation. Panel F. The panel illustrates the difference of the conditional probability distribution 30 min and 48 h after irradiation. Red shades correspond to higher probabilities 30 min post-irradiation while shades of blue indicate larger values 48 h after irradiation. The difference between structures observed 30 min after irradiation and 48 h after irradiation indicate a reversed trend compared to panel D.

The calculation of the conditional probability *p*(*r*|*r*′) shows that the corresponding matrix-representations are strongly diagonal (Fig 8). This is due to the tight clusters of the antibodies marking heterochromatic regions. The structure of the conditional probability matrices supports the previous conclusions. Upon irradiation, the conditional probability (panel B in Fig 8) becomes more homogeneous, indicating a more uniform structure. 48 h after irradiation the conditional probability is again diagonal (panel C in Fig 8). Note that in this case the colour-map has a wider range and values on the diagonal are in fact very close to values from panel A, except for very small radii.

The differences of the conditional probabilities are plotted in Fig 8C–8E. While the changes upon irradiation barely depend on the condition (the value of *r*′), smaller radii were observed to be more abundant before irradiation (red colour in panel C). The difference between the structure 30 min after irradiation and the structure 48 h after irradiation (in panel D, E) indicates an almost uniform and unconditioned increase in the probabilities of the short edges, just as in the case of the unconditioned edge length distribution.

Our results here show that heterochromatic domains undergo structural reorganizations after exposure to ionizing radiation. At 30 min after irradiation the previously very compact and densely organized domains open up and adopt a more loose organization. After 48 h the structures are rehabilitated and the organization approaches again the initial configuration of the non-irradiated cells. In case of 4 Gy irradiation the detected effects were compatible but less pronounced which may be due to a higher amount of double-strand breaks and consequently more repair activity requiring additional space for recruited repair proteins. Moreover, it has to be considered that the percentage of repair activity may be shifted from homologous recombination repair to non-homologous end-joining.

### Euchromatic regions react complementary to heterochromatic regions upon exposure to γ-radiation

**Fig 9:**
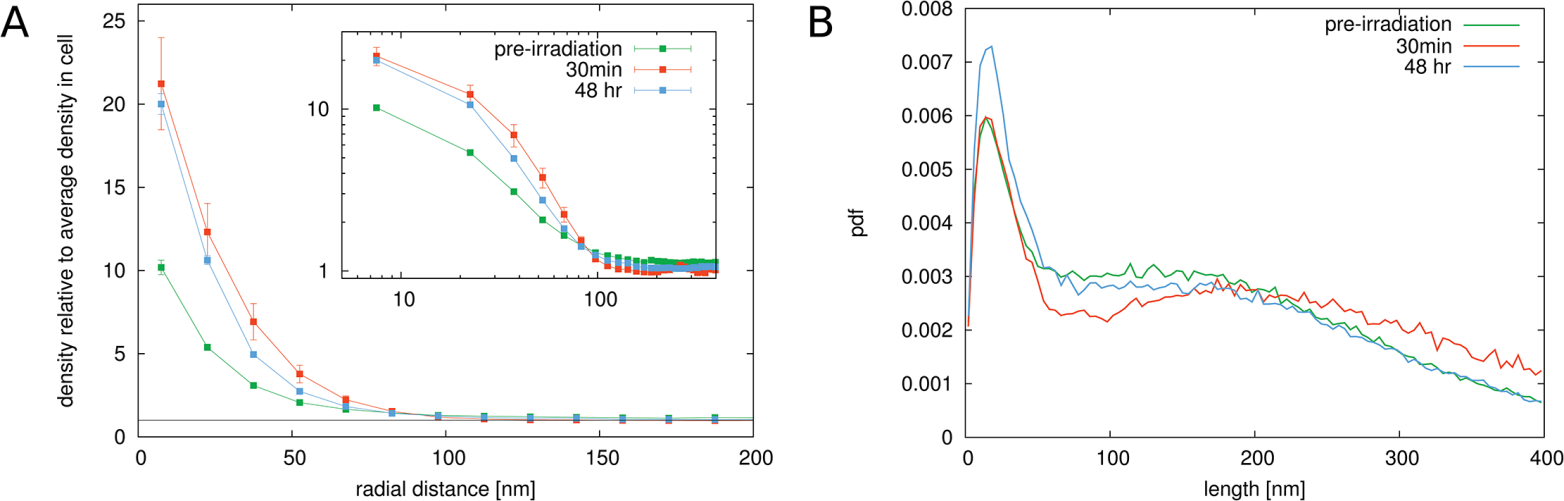
Results for cells exposed to 0.5 Gy radiation. Panel A. The figure shows the radial radial distribution function for H3K4 antibodies representing euchromatin (methylated histone variants) for non-irradiated and irradiated cells. Error bars represent the standard deviation of the mean value after averaging over the sample of cells. The value for *g*(*r*) at small distances goes up to around 20, indicating the high marker densities during repair. The rapid drop off of the radial distribution function within a distance of about 50 nm shows that euchromatin forms small clustered areas. Upon exposure to 0.5 Gy γ-irradiation, a change in the correlation function can be observed in cells that were imaged after 30 min and 48 hrs. The value at small radial distances increases compared to non-irradiated cells. This indicates that the density in the euchromatic regions becomes on average higher in irradiated cells. Panel B. The distribution of edge lengths in the Delaunay triangulation of the markers confirms these observations. A sharp peak in the distribution at around 30 nm can be seen in untreated cells. In 48 h post-irradiation cells, the peak spreads slightly.

Our results for antibodies that are specific for euchromatic regions show that euchromatin responds very differently to irradiation than heterochromatin. The radial distribution function for euchromatic markers is shown in Fig 9 panel A. As for the heterochromatin markers, we obtain high values for the radial distribution function at distances up to 50 nm which means that the markers are also concentrated in small regions. This is expected, since euchromatic regions within the chromosome are organized in specific domains. In cells at 30 minutes after exposure to 0.5 Gy or 4 Gy of ionizing radiation, the value for the radial distribution function is increased compared to non-irradiated cells. Euchromatin appears to show a complementary behaviour to heterochromatin, which was observed to expand. The euchromatic regions seem to condense upon irradiation, possibly due to the expansion of the heterochromatic regions that relocate damage sites into euchromatin [28, 29]. After 48 hours, the structures have slightly recovered. The results of the graph theoretical analysis (Fig 9B) confirm these observations. Upon irradiation, the value of the first peak in the distribution becomes even larger compared to the first minimum indicating a condensation of the euchromatic regions. After 48 hours, the structure of the markers is still not the same as in the beginning. Above all, in contrast to the heterochromatin markers, where the peak was observed to have completely vanished, euchromatin domains undergo a condensation in irradiated cells during repair.

### Cluster analysis independently supports the presented findings

To double-check our observations, we further investigated the behaviour of antibody-position clusters. Two additional sets of cells were prepared and immunolabaled with antibodies for heterochromatin and euchromatin separately. Groups of cells were irradiated using the same radiation source with doses of 0.5 Gy and 3.5 Gy, and 0.5 h, 6 h and 24 h repair times were considered. The data obtained this way was investigated with a simple cluster analysis. Clusters were defined based on the proximity of the localization markers: a point is considered to belong to a given cluster if it has at least three neighbours within a distance of 40 nm. Then, the overall percentage of points in such cluster, compared to the total number of points in a cell, is a good measure of the condensation of the chromatin fibre for the considered markers (for details see [1]).

**Fig 10:**
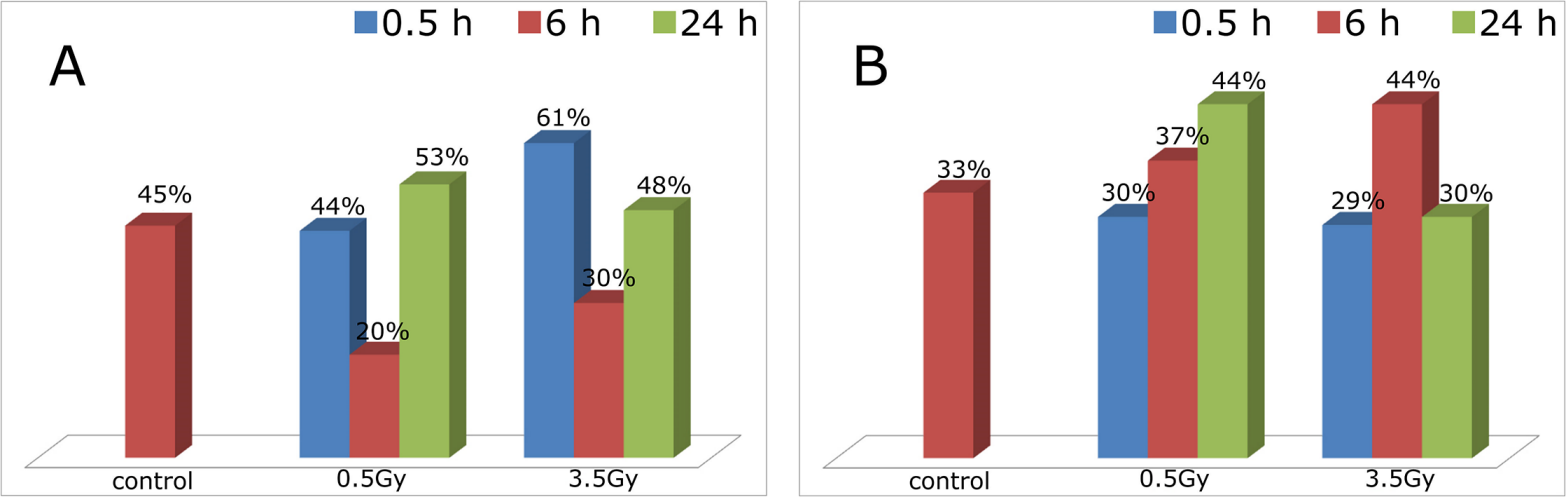
Cluster analysis of the spatial distribution of heterochromatin and euchromatin after irradiation with 0.5 Gy and 3.5 Gy. The analysis was performed on non-irradiated samples, and on samples fixed 6 h and 24 h post irradiation, respectively. **Panel A** present the behaviour of heterochromatic regions irradiated with 0.5 Gy and 3.5 Gy. We observe a decrease in the ratio of the clustered points 6 h after irradiation for both doses. 24 h after irradiation these changes are reverted. This means that a few hours after irradiation the heterochromatin decondenses and after one day the decondensation is reverted. **Panel B** shows the cluster analysis result for euchromatin. These regions behave differently, compared to heterochromatin. For 0.5 Gy, the number of clustered points is increased after 6 h, and the trend continues at the 24 h timestamp. However, for 3.5 Gy, after the initial increase in the percentage of clustered points at 6 h, the ratio drops again in the 24 h measurements. The interpretation of the increase/decrease in the number of clustered points is similar in this case: when the ratio increases, the studied domains condense, when the ratio decreases, the domains relax. This means, that heterochromatin and euchromatin react to irradiation in an opposite way: This analysis, in accordance with the other results, indicates that while heterochromatin opens up soon after irradiation, euchromatin condensates. Given enough time, these processes are reverted in both cases.

Fig 10 shows the results for this analysis. Panel A presents the results for heterochromatin. This shows how the relative number of points in dense clusters decreases 6 h after irradiation and increases again close to the original value 24 h after irradiation. The drop in the number of such points after 6h indicates an opening up of the structure (decondensation), while the increase observed after 24 h corresponds to a recondensation.

For euchromatin, in turn (Panel B), the relative number of such points increases after irradiation. While for 0.5 Gy, the trend continues also after 24 h, for 3.5 Gy the changes observed after 6h are reverted after 24 h. The increase in the number of points in dense clusters at the 6 h timestamp, indicates a condensation of the euchromatin. Similarly, the decrease of the number of such points after 24 h (compared to the 6 h situation) corresponds to a relaxation of the euchromatin domains. In conclusion, the cluster formation in heterochromatin and euchromatin is behaving complementarily.

This separate experiment analysed with a completely different method fully supports our previous observations and result detailed in the sections above.

## Discussion

Many investigations have shown that the detailed study of the genome architecture would offer parameters directly correlated to the chromatin damaging process during irradiation and the following repair [1,2]. Since often all chromatin damage sites cannot be visualized simultaneously, alternative procedures leading to global insights into conformation changes of chromatin are useful but require appropriate methods of quantification. Here we have systematically studied effects obtained from localization microscopy investigations of the nano-architecture of chromatin.

Such studies apply novel approaches in fluorescent light microscopy to circumvent the diffraction limit of light microscopy based on optical separation of individual fluorescent molecules and the determination of their spatial positions. Such techniques are called localization microscopy (for review see [36,37,39]) and have been realized in several slightly different embodiments from which SPDM is one having demonstrated its usefulness in many applications in cell biophysics (for review see [54]). The clear advantages of SPDM are on hand: the use of standard microscopic components, standard preparation techniques and dyes or fluorescent proteins as well as the simultaneous acquisition of wide-field fluorescence images and localization images of the same object without changing the microscopic setup.

Although the typical localization precision and the point-to-point distance resolution of SPDM and other localization microscopy techniques presently is in the order of 10–20 nm especially in biological specimens, the images result in a pointillist presentation which may be incomplete due to limited labelling and detection efficiencies [59]. In addition pointillist images may represent visual local differences of point densities but usually omit any quantitative structural information of the cellular or nuclear environment. This might not be a serious shortcoming in cases where labelled molecules are spatially concentrated to sub-cellular structures which can be clearly separated. In the case of chromatin radiation response we intended to find a global measure over the cell nucleus indicating all architectural changes during the repair process. Since the detected point density appeared to be similar over the entire distribution of nucleosomes, local density fluctuations had to be figured out by advanced techniques of image analysis and calculation. Therefore we applied a novel approach based on statistical physics and graph for the structural non-random chromatin arrangement below 300 nm dimensions. While certain approaches focus on determining the absolute number of nucleosomes [59], we develop a methodology which is independent of the density of these. Nevertheless, the raw data present a reasonable compatibility with the results of Weidemann et al. [59] indicating that the transfection efficiency was still maintained over further cultivation procedures.

The calculation of the distance dependent pair correlation function as well as the application of the graph topological approach of Delaunay triangulation revealed a significant measure for the behaviour of heterochromatin and euchromatin after irradiation and during 48 hours of repair. It indicates that the overall density of antibody positions against heterochromatin becomes smaller in the first fast repair phase (30 min post irradiation) and increases again during the longer repair phase of 48 hours whereas euchromatin behaves complementarily. From these data it can be concluded that heterochromatic regions on average become less compact and the strongly compacted organisation opens up and adopts a more loose structure upon exposure to ionizing radiation. An average drop of 70% of the mean antibody density in a sphere with a radius of 30 nm around an antibody for cells irradiated with 0.5 Gy irradiation after 30 min was observed. Therefore, initial repair of DNA double-strand breaks caused by irradiation in heterochromatin seems to require a drastic decrease of the chromatin density and a strong relaxation of the compact organisation of the chromatin support recent results. 48 h after irradiation, this value is only at around 30% which indicates that structures seem to have recovered after successful repair of the broken DNA strands. Although so far the repair foci (e.g. γH2AX foci) were not analysed (being subject of future investigations), these data support recent findings of others who describe a transfer of break-point foci after X-ray or heavy ion exposure to the border of heterochromatic regions or into euchromatic regions for further repair [2,20,28, 29,69,70]. These findings are also supported by computer simulations of double-strand breaks [71]. However, further effort has to be spent in the systematic analysis of heterochromatin and also euchromatin behaviour after ionizing radiation exposure [Krufczik et al., manuscript in preparation]. Using the same cell line as in our studies, a decondensation of the heterochromatin was observed also in histone acetylation processes on length scales of chromosome territories from 200 nm up to the micrometre scale [58]. These regulatory processes without DNA-breakage, however, are different in nature from the effects of ionizing radiation, which, as our results showed it, appear below 300 nm. Thus, it can be concluded that heterochromatin conformation changes are occurring on different size scales but recover after a certain time of cell activity. This indicates that the architecture on the micro- as well as on the nano-scale remains “ordered in randomness” [72].

The goal of this publication presented here is to demonstrate the power of the applied analysis techniques and to validate them on data sets typical for radiation research. Further investigations will study not only different doses but also different photon spectra at more time points during repair. In addition dynamics of molecular repair foci conformation will be studied. Since the detected effects are not correlated to the kind of damage induced, such data may be useful not only to describe global behaviour of heterochromatin and euchromatin after irradiation but also to obtain a sensitive measure for biological dosimetry at least in the so far difficult to access low dose range.

In this context it may argued that the selection of a 2D image section through the cell nucleus covering only about 1/10 of the nuclear volume may be an incomplete description of the radiation induced re-arrangements in a cell nucleus. From the microscopic side of view this shortcoming could be overcome by the application of 3D-SPDM [49,52]. Although the principle feasibility of this approach using a combination of SPDM and standing wave-field illumination has been demonstrated for cellular imaging, it is very time consuming and extremely data loaded. However, present approaches of large scale data management [73] may improve data handling. On the other hand the data presented here indicate that a 2D section contains enough information for an approach of comparison even if the analyses are restricted to an arbitrary selected part of the nucleus´ volume.

In contrast to the results obtained for heterochromatin, the results of pair correlation functions and Delaunay triangulation for the spatial nucleosomal distribution seem to be rather insensitive either for dose difference or for repair time. At a first glimpse this would not have been expected from all experimental evidence of chromatin re-arrangements and functional cellular activities during repair. On the other hand considering the results of heterochromatin and euchromatin there may be complementary trends in the compaction behaviour of heterochromatin and euchromatin during the repair process so that on average it looks like a compensation of heterochromatin de-compaction and euchromatin increase of compaction. If one would accept this principle behaviour our results could be seen as a validation for this effect.

As recently shown [53] SPDM pointillist images of molecular processes and cellular structures offer differently structured regions by visual inspection which however averages by calculation of density and distance frequency distributions. Therefore more detailed evaluations of sub-regions may be advantageous and/or further sophisticated procedures should be applied. Here the calculation of the conditional probability of the edge lengths of the nearest neighbour graph was tested for the analyses of nucleosomal pattern after radiation exposure.

SPDM imaging as well as many other techniques of localization microscopy [36,37] offer fascinating novel insights into the nano-cosmos of cells and cell nuclei [54,74] and may become key tools for investigations of molecular cellular dynamics and mechanisms behind epigenetic functions. The pointillist highly resolved images contain huge amount of individual information of molecules. The interpretation of the “nicely looking” images in terms of biological relevance and functional mechanisms challenges image analysis and bio-informatics. Depending on the scientific questions sophisticated approaches of quantitative image evaluation and interpretation beyond “nice” visualization have to be tested and validated against optical insufficiencies [37]. Under this perspective our work combining experimental and theoretical biophysics contributes to the elucidation of the so far insufficiently understood repair mechanisms depending on the nano-architecture of chromatin and its dynamics. Further investigations are in progress.

## References

[1] Falk M, Hausmann M, Lukášová E, Biswas A, Hildenbrand G, Davídková M, et al (2014) Giving OMICS spatiotemporal dimensions by challenging microscopy: From functional networks to structural organization of cell nuclei elucidating mechanisms of complex radiation damage response and chromatin repair–PART B (Structuromics). rit. Rev. Eukaryot. Gene Express. 24: 225–247.

[2] Falk M, Lukášová E, Hausmann M, Biswas A, Hildenbrand G, Davídková M, et al (2014) Giving OMICS spatiotemporal dimensions by challenging microscopy: From functional networks to structural organization of cell nuclei elucidating mechanisms of complex radiation damage response and chromatin repair–PART A (Radiomics). Crit. Rev. Eukaryot. Gene Express. 24: 205–223. 25072147

[3] Bolzer A, Kreth G, Solovei I, Koehler D, Saracoglu K, Fauth C, et al (2005) Three-dimensional maps of all chromosomes in human male fibroblast nuclei and prometaphase rosettes. PlosBiol 3: e157 15839726 10.1371/journal.pbio.0030157 PMC1084335

[4] Cremer T, Cremer C (2001) Chromosome territories, nuclear architecture and gene regulation in mammalian cells. Nat Rev Genet 2: 292–301. 11283701 10.1038/35066075

[5] Cremer T, Cremer M, Dietzel S, Müller S, Solovei I, Fakan S (2006) Chromosome territories—a functional nuclear landscape. Curr. Opin. Cell Biol. 18: 307–316. 16687245 10.1016/j.ceb.2006.04.007

[6] Albiez H, Cremer M, Tiberi C, Vecchio L, Schermelleh L, Dittrich S, et al (2006) Chromatin domains and the interchromatin compartment form structurally defined and functionally interacting nuclear networks. Chromos. Res. 14: 707–713. 10.1007/s10577-006-1086-x 17115328

[7] Cavalli G, Misteli T (2013) Functional implications of genome topology.Nat. Struct. Mol. Biol. 20: 290–299. 10.1038/nsmb.2474 23463314 PMC6320674

[8] Lanctôt C, Cheutin T, Cremer M, Cavalli G, Cremer T (2007) Dynamic genome architecture in the nuclear space: regulation of gene expression in three dimensions. Nat. Rev. Genet. 8: 104–115. 17230197 10.1038/nrg2041

[9] Postberg J, Lipps HJ, Cremer T (2010) Evolutionary origin of the cell nucleus and its functional architecture. Essays Biochem. 48: 1–24 10.1042/bse0480001 20822483

[10] Craig JM (2004) Haterochromatin-many flavours, common themes. BioEssays 27: 17–28. 10.1002/bies.20145 15612037

[11] Dimitri P, Caizzi R, Giordano E, Accardo MC, Lattanzi G, Biamonti G (2009) Constitutive heterochromatin: a surprising variety of expressed sequences. Chromosoma 118: 419–435 10.1007/s00412-009-0211-y 19412619

[12] Dimitri P, Corradini N, Rossi F, Verni F (2004) The paradox of functional heterochromatin. BioEssays 27: 29–41 10.1002/bies.20158 15612038

[13] Du Toit A (2012) Chromatin: Defining heterochromatin. Nat Rev Mol Cell Biol 13: 684–685 10.1038/nrm3452 23011340

[14] Eissenberg JC, Elgin SCR (2014) Heterochromatin and Euchromatin In: eLS. John Wiley & Sons Ltd, Chichester http://www.els.net. 10.1002/9780470015902.a0001164.pub3

[15] Foster HA, Bridger JM (2005) The genome and the nucleus: a marriage made by evolution. Genome organisation and nuclear architecture. Chromosoma 114: 212–229. 16133352 10.1007/s00412-005-0016-6

[16] Küpper K, Kölbl A, Biener D, Dittrich S, von Hase J, Thormeyer T, et al (2007) Radial chromatin positioning is shaped by local gene density, not by gene expression. Chromosoma 116: 285–306. 17333233 10.1007/s00412-007-0098-4 PMC2688818

[17] Lukásová E, Kozubek S, Kozubek M, Falk M, Amrichová J (2002) The 3D structure of human chromosomes in cell nuclei. Chromosome Res 10: 535–548. 12498343 10.1023/a:1020958517788

[18] Parada LA, McQueen PG, Munson PJ, Misteli T (2002) Conservation of relative chromosome positioning in normal and cancer cells. Curr Biol 12: 1692–1697. 12361574 10.1016/s0960-9822(02)01166-1

[19] Bekker-Jensen S, Lukas C, Kitagawa R, Melander F, Kastan MB, Bartek J, et al (2006) Spatial organization of the mammalian genome surveillance machinery in response to DNA strand breaks. J. Cell Biol. 173: 195–206. 16618811 10.1083/jcb.200510130 PMC2063811

[20] Falk M, Lukasova E, Kozubek S (2010) Higher order chromatin structure in DSB induction, repair and misrepair. Mutat Res 704: 88–100. 10.1016/j.mrrev.2010.01.013 20144732

[21] Jerabek H, Heermann DW (2012), Expression-Dependent Folding of Interphase Chromatin, PLoS One 7: e37525 10.1371/journal.pone.0037525 22649534 PMC3359300

[22] Monajembashi S, Rapp A, Schmitt E, Dittmar H, Greulich KO, Hausmann M (2005) Spatial association of homologous pericentric regions in human lymphocyte nuclei during repair. Biophys. J. 88: 2309–2322 15626712 10.1529/biophysj.104.048728 PMC1305280

[23] Schwarz-Finsterle J, Scherthan H, Huna A, González P, Müller P, Schmitt E, et al (2013) Volume increase and spatial shifts of chromosome territories in nuclei of radiation-induced polyploidizing tumour cells. Mutat. Res.: Genet. Toxicol. Environ. Mutagen. 756: 56–65 10.1016/j.mrgentox.2013.05.004 23685102

[24] Skalníková M, Kozubek S, Lukásová E, Bártová E, Jirsová P, Cafourková A, et al (2000) Spatial arrangement of genes, centromeres and chromosomes in human blood cell nuclei and its changes during the cell cycle, differentiation and after irradiation. Chromos. Res. 8: 487–499. 10.1023/a:1009267605580 11032319

[25] Wiech T, Stein S, Lachenmaier V, Schmitt E, Schwarz-Finsterle J, Wiech E, et al (2009) Spatial allelic imbalance of BCL2 genes and chromosome 18 territories in nonneoplastic and neoplastic cervical squamous epithelium. Eur. Biophys. J. 38: 793–806 10.1007/s00249-009-0474-5 19495739

[26] Falk M, Lukasova E, Gabrielova B, Ondrej V, Kozubek S (2007) Chromatin dynamics during DSB repair. Biochim Biophys Acta 1773: 1534–1545. 17850903 10.1016/j.bbamcr.2007.07.002

[27] Falk M, Lukasova E, Kozubek S (2008) Chromatin structure influences the sensitivity of DNA to γ-radiation. Biochim Biophys Acta 1783: 2398–2414. 10.1016/j.bbamcr.2008.07.010 18706456

[28] Falk M, Lukášová E, Stefančíková L, Baranová E, Falková I, et al (2013) Heterochromatinization associated with cell differentiation as a model to study DNA double strand break induction and repair in the context of higher-order chromatin structure. Appl. Radiat. Isot.: 10.1016/j.apradiso.2013.01.029 23454236

[29] Jakob B, Splinter J, Conrad S, Voss KO, Zink D, et al (2011) DNA double-strand breaks in heterochromatin elicit fast repair protein recruitment, histone H2AX phosphorylation and relocation to euchromatin. Nucleic Acids Res 39: 6489–6499. 10.1093/nar/gkr230 21511815 PMC3159438

[30] Rouquette J, Cremer C, Cremer T, Fakan S (2010) Functional nuclear architecture studied by microscopy: present and future. Int Rev Cell Mol Biol 282: 1–90. 10.1016/S1937-6448(10)82001-5 20630466

[31] Ježková L, Falk M, Falková I, Davídková M, Bačíková A, Štefančíková L, et al (2014) Function of chromatin structure and dynamics in DNA damage, repair and misrepair: γ-rays and protons in action. Appl. Radiat. Isot.: 10.1016/j.apradiso.2013.01.022 23415104

[32] Girst S, Hable V, Drexler GA, Greubel C, Siebenwirth C, et al (2013) Subdiffusion supports joining of correct ends during repair of DNA double-strand breaks. Scientific reports 3: 2511 10.1038/srep02511 23979012 PMC3753591

[33] Bohn M, Diesinger P, Kaufmann R, Weiland Y, Müller P, Gunkel M, et al (2010) Localization microscopy reveals expression-dependent parameters of chromatin nanostructure. Biophys. J. 99: 1358–1367 10.1016/j.bpj.2010.05.043 20816047 PMC2931727

[34] Abbe E (1873) Beiträge zur Theorie des Mikroskops und der mikroskopischen Wahrnehmung (Contributions to the theory of the microscope and microscopic observation). Archiv f. mikroskopische Anatomie 9: 411–468.

[35] Rayleigh L (1896) On the theory of optical images, with special reference to the microscope. Philos Mag 42: 167–195.

[36] Cremer C, Masters BR (2013) Resolution enhancement techniques in microscopy. Eur Phys J H38: 281–344.

[37] Deschout H, Zanacchi FC, Mlodzianoski M, Diaspro A, Bewersdorf J, Hess ST, et al (2014) Precisely and accurately localizing single emitters in fluorescence microscopy. Nat Meth 11: 253–266. 10.1038/nmeth.2843 24577276

[38] Schermelleh L, Heintzmann R, Leonhardt H (2010) A guide to super-resolution fluorescence microscopy. J Cell Biol 190: 165–175. 10.1083/jcb.201002018 20643879 PMC2918923

[39] Cremer C, Kaufmann R, Gunkel M, Pres S, Weiland Y, Müller P, et al (2011) Superresolution imaging of biological nanostructures by spectral precision distance microscopy. Biotechnol J 6:1037–1051. 10.1002/biot.201100031 21910256

[40] Huang B, Wang W, Bates M, Zhuang X (2008) Three-Dimensional Super-Resolution Imaging by Stochastic Optical Reconstruction Microscopy. Science 319: 810–813. 10.1126/science.1153529 18174397 PMC2633023

[41] Esa A, Edelmann P, Kreth G, Trakhtenbrot L, Amariglio N, Rechavi G, et al (2000) Three-dimensional spectral precision distance microscopy of chromatin nano-structures after triple-colour DNA labelling: a study of the BCR region on chromosome 22 and the Philadelphia chromosome. J Microsc 199: 96–105. 10947902 10.1046/j.1365-2818.2000.00707.x

[42] Heilemann M, Herten DP, Heintzmann R, Cremer C, Mueller C, Tinnefeld P, et al (2002) High-resolution colocalization of single dye molecules by fluorescence lifetime imaging microscopy. Analyt Chem 74: 3511–3517. 12139062 10.1021/ac025576g

[43] Betzig E, Patterson GH, Sougrat R, Lindwasser OW, Olenych S, Bonifacino J, et al (2006) Imaging intracellular fluorescent proteins at nanometer resolution. Science 313:1642–1645. 16902090 10.1126/science.1127344

[44] Hess ST, Girirajan TP, Mason MD (2006) Ultra-high resolution imaging by fluorescence photoactivation localization microscopy. Biophys J 91:4258–4272. 16980368 10.1529/biophysj.106.091116 PMC1635685

[45] Rust MJ, Bates M, Zhuang X (2006) Sub-diffraction-limit imaging by stochastic optical reconstruction microscopy (STORM). Nature Meth 3: 793–795. 10.1038/nmeth929 PMC2700296 16896339

[46] Lemmer P, Gunkel M, Baddeley D, Kaufmann R, Urich A, Weiland Y, et al (2008) SPDM–light microscopy with single molecule resolution at the nanoscale. Appl Phys B 93: 1–12.

[47] Lemmer P, Gunkel M, Weiland Y, Müller P, Baddeley D, Kaufmann R, et al (2009) Using conventional fluorescent markers for far-field fluorescence localization nanoscopy allows resolution in the 10 nm range. J Microsc 235: 163–171. 10.1111/j.1365-2818.2009.03196.x 19659910

[48] Fölling J, Bossi M, Bock H, Medda R, Wurm C, Hein B, et al (2008) Fluorescence nanoscopy by ground-state depletion and single-molecule return. Nature Methods 5: 943–945. 10.1038/nmeth.1257 18794861

[49] Kaufmann R, Lemmer P, Gunkel M, Weiland Y, Müller P, Hausmann M, et al (2009) SPDM–Single molecule superresolution of cellular nanostructures. Proc. SPIE 7185: 71850J1–71850J19 10.1117/12.809109

[50] Hendrix J, Flors C, Dedecker P, Hofkens J, Engelborghs Y (2008) Dark states in monomeric red fluorescent proteins studied by fluorescence correlation and single molecule spectroscopy. Biophys J 94: 4103–4113. 10.1529/biophysj.107.123596 18234806 PMC2367191

[51] Sinnecker D, Voigt P, Hellwig N, Schaefer M (2005) Reversible photobleaching of enhanced green fluorescent proteins. Biochemistry 44: 7085–7094. 15865453 10.1021/bi047881x

[52] Kaufmann R, Müller P, Hildenbrand G, Hausmann M, Cremer C (2011) Analysis of Her2/neu membrane protein clusters in different types of breast cancer cells using localization microscopy. J Microsc 242: 46–54. 10.1111/j.1365-2818.2010.03436.x 21118230

[53] Müller P, Lemmermann NA, Kaufmann R, Gunkel M, Paech D, Hildenbrand G, et al (2014) Spatial distribution and structural arrangement of a murine cytomegalovirus glycoprotein detected by SPDM localization microscopy. Histochem. Cell Biol. 142: 61–67. 10.1007/s00418-014-1185-2 24504601

[54] Müller P, Weiland Y, Kaufmann R, Gunkel M, Hillebrandt S, Cremer C, et al. (2012) Analysis of fluorescent nanostructures in biological systems by means of Spectral Position Determination Microscopy (SPDM). In: “Current microscopy contributions to advances in science and technology” (Méndez-Vilas A, ed.), Vol. 1: 3–12.

[55] Baddeley D, Cannell MB, Soeller C (2010) Visualization of localization microscopy data. Microsc Microanal 16: 64–72. 10.1017/S143192760999122X 20082730

[56] Kaufmann R, Piontek J, Grüll F, Kirchgessner M, Rossa J, Wolburg H, et al (2012) Visualization and quantitative analysis of reconstituted tight junctions using localization microscopy. PLoS One 7: e31128 10.1371/journal.pone.0031128 22319608 PMC3271094

[57] Knoch TA (2002) Approaching the Three-Dimensional Organization of the Human Genome Heidelberg, TAK Press, 3-00-009959-X.

[58] Fejes-Toth KF, Knoch TA, Wachsuth M, Frank-Stohr M, Stohr M, Bacher CP, et al (2004) Trichostatin A-induced histone acetylation causes decondensation of interphase chromatin. Cell Sci. 117: 4277–87. 15292402 10.1242/jcs.01293

[59] Weidemann T, Wachsmuth M, Knoch TA, Mueller G, Waldeck W, Langowski J (2003) Counting Nucleosomes in Living Cells with a Combinaiton of Fluorescence Correlation Spectroscopy and Confocal Imaging. J. Mol. Biol, 334, s.l.: pp. 229–240. 14607115 10.1016/j.jmb.2003.08.063

[60] Scott DW (1992) Multivariate densitz estimation, Wiley, New York, ISBN 0471547700

[61] West DB (2000) Introduction to Graph Theory, Prentice Hall, ISBN 0130144002

[62] Bajardi P, Poletto C, Ramasco JJ, Tizzoni M, Colizza V, Vespignani A (2011) Human mobility networks, travel restrictions, and the global spread of 2009 h1n1 pandemic. PLoS ONE, 6(1):e16591 10.1371/journal.pone.0016591 21304943 PMC3031602

[63] Buldyrev SV, Parshani R, Paul G, Stanley HE, Havlin S (2010) Catastrophic cascade of failures in interdependent networks. Nature, 464(7291):1025–1028. 10.1038/nature08932 20393559

[64] O’Roak BJ, Vives L, Girirajan S, Karakoc E, Krumm N, Coe BP, et al (2012) Sporadic autism exomes reveal a highly interconnected protein network of de novo mutations. Nature, 485(7397):246–250. 10.1038/nature10989 22495309 PMC3350576

[65] Vespignani A (2012) Modelling dynamical processes in complex sociotechnical systems. Nat Phys, 8(1):32–39

[66] Vinayagam A, Stelzl U, Foulle R, Plassmann S, Zenkner M, Timm J, et al (2011) A directed protein interaction network for investigating intracellular signal transduction. Science Signaling, 4(189):rs8 10.1126/scisignal.2001699 21900206

[67] Delaunay B (1934) Sur la sphere vide. Izvestia Akademii Nauk SSSR, Otdelenie Matematicheskikh i Estestvennykh Nauk, 6: 793–800.

[68] Aurenhammer F (1991) Voronoi diagrams–a survey of a fundamental geometric data structure. Acm Computing Surveys, 23(3):345–405

[69] Jakob B, Splinter J, Durante M, Taucher-Scholz G (2009) Live cell microscopy analysis of radiation-induced DNA double-strand break motion. Proc Natl Acad Sci U S A 106: 3172–3177. 10.1073/pnas.0810987106 19221031 PMC2642473

[70] Jakob B, Splinter J, Taucher-Scholz G (2009) Positional stability of damaged chromatin domains along radiation tracks in mammalian cells. Radiat Res 171: 405–418. 10.1667/RR1520.1 19397441

[71] Zhang Y, Heermann DW (2014) DNA Double-Strand Breaks: Linking Gene Expression to Chromosome Morphology and Mobility. Chromosoma, 123(1–2): 103–15 10.1007/s00412-014-0480-y 23982751

[72] Kozubek S, Lukásová E, Jirsová P, Koutná I, Kozubek M, et al (2002) 3D Structure of the human genome: order in randomness. Chromosoma 111: 321–331. 12474061 10.1007/s00412-002-0210-8

[73] Grunzke R, Hesser J, Starek J, Kepper N, Gesing S, Hardt M, et al. (2014) Device-driven metadate management solution for scientific big data use cases. 22nd Euromicro Int. Conf. Parallel, Distributed, and Network-Based Processing (PDP 2014), February 2014, Turin, Italy. IEEE Comp. Soc. Proc. PDP 2014: 317–321 (doi: 10.1109/PDP.2014. 119)

[74] Hausmann M, Müller P, Kaufmann R, Cremer C (2013) Entering the nano-cosmos of the cell by means of spatial position determination microscopy (SPDM): Implications for medical diagnostics and radiation research. IFMBE Proc 38: 93–95.

